# Associations between polygenic risk of substance use and use disorder and alcohol, cannabis, and nicotine use in adolescence and young adulthood in a longitudinal twin study

**DOI:** 10.1017/S0033291721004116

**Published:** 2023-04

**Authors:** Jonathan D. Schaefer, Seon-Kyeong Jang, D. Angus Clark, Joseph D. Deak, Brian M. Hicks, William G. Iacono, Mengzhen Liu, Matt McGue, Scott I. Vrieze, Sylia Wilson

**Affiliations:** 1 Institute for Child Development, University of Minnesota, Minneapolis, MN, USA; 2 Department of Psychology, University of Minnesota, Minneapolis, MN, USA; 3 Department of Psychiatry, University of Michigan, Ann Arbor, MI, USA; 4 Department of Psychiatry, Yale University School of Medicine, New Haven, CT, USA; 5 Department of Psychiatry, Veterans Affairs Connecticut Healthcare Center, West Haven, CT, USA

**Keywords:** Alcohol, behavioral genetics, cannabis, disinhibition, family history, longitudinal, nicotine, polygenic risk, substance use disorder, twin

## Abstract

**Background:**

Recent well-powered genome-wide association studies have enhanced prediction of substance use outcomes via polygenic scores (PGSs). Here, we test (1) whether these scores contribute to prediction over-and-above family history, (2) the extent to which PGS prediction reflects inherited genetic variation *v.* demography (population stratification and assortative mating) and indirect genetic effects of parents (genetic nurture), and (3) whether PGS prediction is mediated by behavioral disinhibition prior to substance use onset.

**Methods:**

PGSs for alcohol, cannabis, and nicotine use/use disorder were calculated for Minnesota Twin Family Study participants (*N* = 2483, 1565 monozygotic/918 dizygotic). Twins' parents were assessed for histories of substance use disorder. Twins were assessed for behavioral disinhibition at age 11 and substance use from ages 14 to 24. PGS prediction of substance use was examined using linear mixed-effects, within-twin pair, and structural equation models.

**Results:**

Nearly all PGS measures were associated with multiple types of substance use independently of family history. However, most within-pair PGS prediction estimates were substantially smaller than the corresponding between-pair estimates, suggesting that prediction is driven in part by demography and indirect genetic effects of parents. Path analyses indicated the effects of both PGSs and family history on substance use were mediated via disinhibition in preadolescence.

**Conclusions:**

PGSs capturing risk of substance use and use disorder can be combined with family history measures to augment prediction of substance use outcomes. Results highlight indirect sources of genetic associations and preadolescent elevations in behavioral disinhibition as two routes through which these scores may relate to substance use.

## Introduction

Substance use disorders (SUDs) are one of the most common and disabling behavioral health problems (Whiteford et al., 2013), with lifetime prevalence estimates from longitudinal studies coalescing around 40% (Angst et al., 2016; Hamdi & Iacono, 2014; Schaefer et al., 2017). A recent influx of well-powered genome-wide association studies (GWASs) has led to substantial increases in the ability to predict substance use and use disorder via polygenic scores (PGSs). PGSs place individuals somewhere along a continuum of risk by taking the sum of the total number of trait-associated alleles across the genome, weighted by risk allele effect sizes from the corresponding GWASs (Choi, Mak, & O'Reilly, 2020). Although these scores generally explain only a few percent of the variance in their respective phenotypes, PGSs have been used for a range of purposes in the biomedical and social sciences to date, including identifying shared etiology among traits, testing for genome-wide gene-by-environment interaction, and – when combined with other clinical and demographic information – patient stratification (Hoffmann et al., 2017; Torkamani, Wineinger, & Topol, 2018; Wray et al., 2018).

Despite increasing interest in these measures, the clinical utility of PGSs capturing risk of substance use/SUD is constrained by several factors. One is that it is unclear whether PGSs contribute to the prediction of these phenotypes over-and-above family history, which can be less expensive and easier to obtain. Family history is frequently used as a risk indicator in the studies of substance use due to the observation that children with a family history of SUDs are more likely to experience their own substance-related problems than children with no family history, even when raised by non-disordered adoptive parents (Kendler et al., 2012, 2015).

A second limitation is that the mechanisms by which polygenic risk of substance use/SUDs relate to phenotypes are largely unknown. Recent research suggests that, in addition to direct genetic effects (i.e. attributable to inherited genetic variation), PGSs also predict trait variance via demographic processes (i.e. population stratification and assortative mating) (Trejo & Domingue, 2018). One additional possibility is that parents generate family environments consistent with their own genotypes (e.g. characterized by low-parental monitoring), which facilitate the development of substance use problems in offspring, thus inducing a correlation between offspring genotype and family environment that is environmentally mediated (Kong et al., 2018). A powerful approach to controlling for these indirect genetic effects involves the use of sibling comparisons. Siblings raised in the same family share all familial genetic influences that shape their environment, and potential bias due to assortative mating and population stratification are eliminated in within-family estimates (Brumpton et al., 2020). Consequently, within-family PGS analyses account for indirect effects related to common family environments. The use of dizygotic (DZ) co-twins strengthens this approach further by ensuring similar timing of shared environmental influences (e.g. socioeconomic status and parent age) between twins.

It is commonly observed that PGSs derived based on the use of a specific substance (e.g. alcohol) tend to predict both the use of that and other substances (e.g. cannabis and nicotine) (Chang et al., 2019; Deak et al., 2020). Thus, these putatively substance-specific scores may also capture risk mechanisms that increase the severity of multiple types of substance use simultaneously. One promising candidate is behavioral disinhibition, an early-emerging personality trait defined by difficulty with inhibiting impulses to behave in socially undesirable ways (Iacono, Malone, & McGue, 2008). Behavioral disinhibition is a plausible mediator in this context because it (1) is heritable and evident in childhood, prior to substance use onset (Hicks, Foster, Iacono, & McGue, 2013; Tuvblad, May, Jackson, Raine, & Baker, 2017; Young, Stallings, Corley, Krauter, & Hewitt, 2000), (2) tends to be higher among children with a positive family history of substance disorder (Handley et al., 2011; King et al., 2009), and (3) has robust, prospective associations with several forms of externalizing psychopathology (Mezquita, Ibáñez, Moya, Villa, & Ortet, 2014; Quay, 1997), including SUDs (Sher, Bartholow, & Wood, 2000; Wilson, Malone, Venables, McGue, & Iacono, 2021).

The aims of the current study are thus threefold. First, we aimed to test whether PGS and family history measures of substance use/SUD risk are independently associated with multiple types of substance use. Second, we conducted within-twin pair analyses of PGSs and substance use to establish the extent to which PGS prediction of substance use is attributable to the effects of demography and indirect effects of parents. Third, we tested the hypothesis that the non-specific associations observed between risk indicators and substance use are mediated by elevated behavioral disinhibition in preadolescence. To achieve these aims, we used data from a longitudinal study of twins followed from age 11 to adulthood, with family history, genotype, and behavioral disinhibition assessed at baseline and alcohol, cannabis, and nicotine use assessed between ages 14 and 24 years.

## Methods

### Participants and procedures

Participants were drawn from the Minnesota Twin Family Study (MTFS), a longitudinal study of same-sex twins [*N* *=* 2510 (1578 monozygotic (MZ)/932 DZ), 49.0% male]. To be eligible for the study, participants had to reside within a day's drive of Minneapolis, live with at least one biological parent, and have no physical or mental conditions that would interfere with completing a day-long, in-person assessment. Participants were recruited the year they turned 11 years old between 1977 and 1984 or 1988 and 1994. Families were representative of the area they were drawn from in terms of socioeconomic status, history of mental health treatment, and urban *v.* rural residence. Consistent with the demographics of Minnesota for the target birth years, 96% of participants reported non-Hispanic White race and ethnicity. Detailed overviews of the MTFS, twin samples, inclusion/exclusion criteria, and assessment procedures are provided in previous studies (Iacono, Mcgue, & Krueger, 2006; Keyes et al., 2009; Wilson et al., 2019). All participants completed an informed consent/assent process (parental consent for their own participation and that of their children under 18 years, twin assent before 18 years and consent after 18 years).

Study participants and their parents were first assessed at age 11 (*M*_age_ = 11.8 years, s.d. = 0.4 years). At this assessment, participants and parents were interviewed separately by different interviewers regarding substance use, personality traits, and mental disorders. Interviewers had at least a bachelor-level degree in psychology and went through extensive training. Participants, mothers, and teachers also completed measures reporting on the adolescent participants' behavior. Participants were assessed repeatedly for substance use every 4 to 7 years from age 14 (*M*_age_ = 14.9, s.d. = 0.6 years) to age 24 (*M*_age_ = 25.0, s.d. = 0.9 years; >85% retention rate). To ensure that measures of behavioral disinhibition at age 11 were not influenced by early substance use, we excluded twins who endorsed any history of substance use at their baseline assessment (*n* = 27) from analysis.

### Measures

#### Risk indicators

*Polygenic scores* (*PGSs*). We calculated PGSs capturing risk of alcohol, cannabis, and nicotine use/use disorder using summary statistics from six recently published GWASs, summarized in Table 1. Genotyping procedures used in MCTFR have been described previously (Miller et al., 2013). Briefly, participants were genotyped on an Illumina 660W-Quad microarray (Illumina, Inc., San Diego, CA). Imputation was conducted using the Haplotype Reference Consortium panel on the Michigan imputation server (Das et al., 2016; The Haplotype Reference Consortium, 2016). We selected only twins of primarily European ancestry for polygenic scoring. To identify these individuals, we calculated four principal components (PCs) for the European sample in the 1000 Genomes Project Consortium (1000G) (Auton et al., 2015), scored MTFS genotypes with these 1000G PC weights using PLINK1.9 (Chang et al., 2015), and selected twins falling within the boundaries of the four 1000G European PCs.

**Table 1.** Details of the GWASs that generated the weights used to calculate PGSs in the MTFS cohort

Polygenic score	Citation	Assessment of target phenotype	Discovery sample size	*h*^2^_SNP_ (%)
Drinks per week	Liu et al. (2019)	Average number of drinks a participant reported drinking each week, aggregated across all types of alcohol	941 280	4
Problematic alcohol use	Zhou et al. (2020)	Lifetime diagnosis of alcohol use disorder (ICD) or alcohol dependence (DSM-IV); scores on a measure of problematic drinking (AUDIT-P)	435 563	6–11
Lifetime cannabis use	Pasman et al. (2018)	Self-reported lifetime use of cannabis	184 765	11
Cannabis use disorder	Johnson et al. (2020)	Lifetime diagnosis of cannabis use disorder (DSM-5) or cannabis abuse/dependence (DSM-IV, DSM-III, and ICD-10)	384 032	6–12
Regular smoking	Liu et al. (2019)	Self-reported regular smoking	1 232 091	8
Nicotine dependence	Quach et al. (2020)	Scores on the Fagerstrom Test for Nicotine Dependence (categorized as mild, moderate, or severe)	58 000	9

*h*^2^_SNP_, SNP heritability, defined as the fraction of the phenotypic variance explained by the additive effects of all genotyped SNPs; ICD, International Classification of Diseases; DSM, Diagnostic and Statistical Manual of Mental Disorders; AUDIT-P, Alcohol Use Disorders Identification Test, alcohol-related problem items.

Prior to estimating each score, we conducted the following quality-control procedures: (1) extracted variants from the European-ancestry subset of HapMap3, as these variants are well-characterized; (2) removed indels, multi-allelic sites, variants falling within the MHC region (chr6: 28477797–33448354), and those having a minor allele frequency less than 0.01; and (3) pruned MTFS genotypes to the variants with imputation quality (*R*^2^) greater than 0.7. Polygenic scoring was conducted used LDPred v.1.0.11, a Bayesian method that estimates posterior mean effect sizes from GWAS summary statistics conditioning on a prior defining the genetic architecture of a trait of interest and linkage disequilibrium information from the reference sample (Vilhjálmsson et al., 2015). In cases where the MTFS contributed data to a given GWAS, we requested new summary statistics from the authors calculated with MTFS participants excluded. Based on an infinitesimal model of complex traits (Boyle, Li, & Pritchard, 2017), we assumed the proportion of causal variants in the LDPred model to be 1. We standardized each PGS to a mean of 0 and standard deviation (s.d.) of 1.

*Family history*. Adolescent participants' family history of SUD was determined using parents' self-report of their own lifetime symptoms, collected at the baseline assessment using the expanded Substance Abuse Module (SAM) of the Composite International Diagnostic Interview (CIDI) (Robins, Baber, & Cottler, 1987, 1988). Parents were assigned lifetime alcohol use disorder, cannabis use disorder, or nicotine dependence diagnoses if they endorsed two or more DSM-III-R symptoms (the diagnostic system when the study began) of alcohol abuse or dependence, cannabis abuse or dependence, or nicotine dependence, respectively, consistent with current DSM-5 criteria.^†^^1^ Adolescent participants' family history of alcohol use disorder, cannabis use disorder, and nicotine dependence were thus each coded as ‘1’ if at least one parent met criteria for the respective lifetime diagnosis and coded as ‘0’ if neither parent met lifetime criteria.

#### Substance use

*Substance use indices*. Substance use in each cohort was assessed at ages 11, 14, 17, 20, and 24 years using either a computerized substance use inventory, the SAM of the CIDI (Robins et al., 1987, 1988), or both measures. We computed use indices for alcohol, cannabis, and nicotine use between ages 14 and 24 using twins' responses to items assessing the use of each substance at each wave. Because responses to these items were skewed and tended to cluster around influential values (e.g. ‘100 uses’), we derived ordinal scales from these questions, which were averaged together to provide an index of substance use severity at each wave. We, then, derived indices of overall use for each substance by averaging these index scores across assessment waves.

Alcohol use at each wave was assessed using twins' mean scores on four items: (1) frequency of drinking, (2) number of drinks typically consumed per occasion, (3) maximum number of drinks consumed in 24 h, and (4) misuse (drinking to intoxication). Cannabis use at each wave was estimated using twins' mean scores on items assessing (1) frequency of use and (2) number of uses. Nicotine use at each wave was estimated using twins' scores on items reporting cigarettes used per day adjusted for non-daily use and, when applicable, equivalent use of other tobacco products (e.g. cigars, pipes, and chews). See online Supplementary Table S1 for further details.

Because our three substance use indices had a mean correlation of *r* = 0.57 (range = 0.54–0.61), we used confirmatory factor analysis to derive a single factor capturing substance use between ages 14 and 24. Standardized factor loadings were all positive and significant (alcohol = 0.76, cannabis = 0.71, and nicotine = 0.80; all *p*s < 0.001). We saved factor scores from this just-identified model for use in subsequent analyses.

#### Behavioral disinhibition

Participants' behavioral disinhibition was assessed at age 11 using a multi-method, multi-informant approach, combining data from the following measures:

*Delinquent behavior inventory*. The delinquent behavior inventory is a 36-item self-report measure that inquires about various antisocial acts, such as cutting class, stealing, and getting into fights (*α* = 0.95) (Taylor, McGue, Iacono, & Lykken, 2000). Participants were asked to mark each behavior they had ever engaged in. We used a count of delinquent behaviors endorsed.

*Externalizing disorder symptoms*. Participants' symptoms of attention-deficit hyperactivity disorder, conduct disorder, and oppositional defiant disorder were assessed using the Diagnostic Interview for Children and Adolescent – Revised (DICA-R) (all kappa reliabilities >0.74) (Reich & Welner, 1988). Symptoms were considered present if endorsed either by the participant or the participants' mother. We used a count of these symptoms.

*Teacher-rated externalizing problems*. Ratings of the participants' behavior at school were collected from up to four teachers nominated by the twin and his/her parents. Minnesota had a policy of placing members of a twin pair in different classrooms whenever possible, minimizing potential teacher rating biases. Teachers completed 28 items rating how characteristic different externalizing behavior problems (e.g. ‘often acts without thinking’, ‘has difficulty remaining seated when required to do so’, ‘often actively defies or refuses adult requests or rules’) were of each twin, compared to the average child, on a 4-point scale (1 = not at all, 2 = just a little, 3 = pretty much, 4 = very much). We used the total externalizing problem score (*α* = 0.97), averaged first across items and then across teachers.

Our measures of behavioral disinhibition had a mean correlation of *r* = 0.34 (range = 0.25–0.47). Consequently, we used confirmatory factor analysis to derive a single factor representing behavioral disinhibition at age 11. Standardized factor loadings were all positive and significant (delinquent behavior inventory = 0.38, externalizing disorder symptoms = 0.71, and teacher-reported externalizing problems = 0.66; all *p*s < 0.001), consistent with our conceptualization that each measure taps into a single underlying construct. We saved factor scores from this just-identified model for use in subsequent analyses.

### Statistical analyses

We performed linear mixed-effect, within-twin pair, and structural equation models. Given the limited number of independent tests, results from all models are presented without correction for multiple testing. Generalized linear mixed-effects models comparing twins with present *v.* missing family history, genotype, and behavioral disinhibition data indicated that these groups were statistically comparable in terms of their scores on our substance use factor (*β*s = 0.00–0.29, all *p*s > 0.16).

We first conducted separate linear mixed-effects models examining associations between each of our risk indicators (i.e. PGSs and family history) and substance use indices. We then repeated PGS models adjusting for the corresponding family history variable (e.g. for family history of nicotine dependence in models predicting nicotine use) to test whether PGSs and family history were independently associated with risk.

Second, we used within-twin pair models conducted using DZ twins to estimate the direct genetic effects of each PGS, controlling for demography and the indirect genetic effects of parents.^2^ Briefly, these analyses decompose associations with each PGS into between-pair and within-pair effects (Begg & Parides, 2003; McGue, Malone, Keyes, & Iacono, 2014). The between-pair effect represents the expected change in substance use given a one-unit change in the twin pair PGS mean, whereas the within-pair effect represents the expected change given a one-unit change in the difference between an individual twin's PGS and the twin pair PGS mean. By including both estimates in the same model, each estimate is adjusted for and independent of the other (McGue et al., 2014). In this context, a reduced within-pair PGS prediction estimate relative to the between-pair estimate suggests that the between-pair association is mediated by some combination of demographic and indirect genetic factors. Similar within-pair and between-pair estimates, in contrast, suggest that the between-pair associations are more likely attributable to the direct effects of inherited genetic variation.

Third, we used linear mixed-effects and structural equation models to test whether the nonspecific associations between risk indicators (i.e. PGSs and family history) and multiple types of substance use might be mediated by differences in behavioral disinhibition prior to substance use onset. We initially estimated separate models for each risk indicator to examine whether mediation was consistently observed across measures. Next, given the weak to moderate correlations between many of our risk indicators (see online Supplementary Fig. S1), we estimated a combined model to see which indicators made independent contributions to risk.

Linear mixed-effects models were conducted in R Studio version 1.2.5019 using ‘lmer’ from the ‘lme4’ package with denominator degrees of freedom adjusted using the Kenward–Roger approximation from the ‘lmerTest’ package (Bates, Mächler, Bolker, & Walker, 2015; Kuznetsova, Brockhoff, & Christensen, 2017). The lmer function fits these models using restricted maximum likelihood and listwise deletion of missing data. Random intercepts were included at the twin-pair level to account for within-pair correlations. ‘Behavioral disinhibition’ and ‘substance use’ in these models were represented using factor scores saved from the confirmatory factor models described above. We empirically tested the statistical difference between within-pair and between-pair effects using likelihood ratio tests in Mplus version 8.4. Mediation models were also fit in Mplus using full information maximum likelihood estimation, with confidence intervals (CIs) derived via clustered (by family) nonparametric percentile bootstrap (10 000 repetitions). In these analyses, we embedded the measurement models for ‘behavioral disinhibition’ and ‘substance use’ factors in the broader structural equation models. All models included sex, zygosity, age at the time of outcome assessment, and birth year as covariates. Models including PGSs also included the first 10 genetic PCs.

## Results

Descriptive data for our measures are presented in Table 2. Rates of any substance use between ages 14 and 24 were 94.1, 58.9, and 66.6% for alcohol, cannabis, and nicotine, respectively. Family history of SUDs was also common in our sample, with 62, 36, and 63% of participants with parent data having at least one parent who met criteria for a lifetime alcohol use disorder, cannabis use disorder, or nicotine dependence diagnosis, respectively. Within-pair PGS correlations were all close to expectations (range *r* = 0.46–0.53), given that the expected shared additive genetic variance between siblings is 50% of the total additive genetic variance.

**Table 2.** Descriptive statistics and twin correlations for risk indicator variables, behavioral disinhibition mediator variables, and substance use outcome variables

	*N*	Mean (s.d.)	Possible range	Sample range	rMZ (95% CI)	rDZ (95% CI)
Risk indicators						
Family history						
of substance use disorder	2400	0.80 (0.40)	0–1	0–1	–	–
of alcohol use disorder	2327	0.62 (0.49)	0–1	0–1	–	–
of cannabis use disorder	2297	0.36 (0.48)	0–1	0–1	–	–
of nicotine dependence	2372	0.63 (0.48)	0–1	0–1	–	–
Polygenic scores						
Drinks per week-PGS	2053	0.00 (1.00)	–	–	–	0.49 (0.41–0.57)
Problematic alcohol use-PGS	2053	0.00 (1.00)	–	–	–	0.52 (0.44–0.59)
Lifetime cannabis use-PGS	2053	0.00 (1.00)	–	–	–	0.50 (0.42–0.58)
Cannabis use disorder-PGS	2053	0.00 (1.00)	–	–	–	0.53 (0.44–0.60)
Regular smoking-PGS	2053	0.00 (1.00)	–	–	–	0.46 (0.37–0.54)
Nicotine use disorder-PGS	2053	0.00 (1.00)	–	–	–	0.50 (0.42–0.58)
Behavioral disinhibition, age 11						
Delinquent behavior inventory	2290	1.28 (2.26)	0–36	0–36	0.30 (0.23–0.37)	0.33 (0.24–0.41)
Externalizing disorder symptoms	2483	3.99 (4.41)	0–35	0–25	0.76 (0.72–0.79)	0.42 (0.35–0.50)
Teacher-reported externalizing problems	2269	1.39 (0.45)	1–4	1–3.85	0.76 (0.72–0.79)	0.44 (0.35–0.51)
Disinhibition factor	2098	0.00 (1.00)	–	–	0.80 (0.76–0.82)	0.47 (0.38–0.54)
Cumulative substance use, ages 14–24						
Alcohol use	2418	1.60 (0.94)	0–5.75	0–5	0.80 (0.78–0.83)	0.60 (0.54–0.66)
Cannabis use	2435	0.65 (0.93)	0–5	0–4.75	0.74 (0.71–0.78)	0.52 (0.45–0.58)
Nicotine use	2418	0.89 (1.01)	0–4	0–4	0.75 (0.72–0.78)	0.55 (0.48–0.61)
Substance use factor	2417	0.00 (1.00)	–	–	0.82 (0.80–0.85)	0.61 (0.55–0.67)

s.d., standard deviation; rMZ, monozygotic twin correlations; rDZ, dizygotic twin correlations; CI, confidence interval.

### Do PGSs capturing risk of substance use/SUDs contribute to the prediction of substance use in adolescence and young adulthood over-and-above family history of SUD?

Consistent with prior research, higher scores on each PGS were associated with both an increased use of the substance used to derive the indicator (e.g. cannabis for the Lifetime Cannabis Use-PGS) and use of other substances (e.g. alcohol and nicotine). The only exceptions to this pattern were the Drinks Per Week-PGS, which was not significantly associated with cannabis use, and the Nicotine Dependence-PGS, which was significantly associated with nicotine use only (online Supplementary Table S3). Associations between family history measures and substance use indices (mean *β* = 0.32, range = 0.21–0.47) were all significant and consistently larger than the corresponding associations with PGSs (mean *β* *=* 0.09, range = 0.00–0.20). All significant associations between PGSs and substance use indices in these initial, single-indicator models remained significant after adjusting for family history, suggesting that both PGSs and family history each made independent contributions to predicting substance use (Table 3).

**Table 3.** Associations between each PGS and substance use controlling for family history of alcohol use disorder, cannabis use disorder, and/or nicotine dependence

Predictors	Cumulative substance use indices ages 14–24							
	Alcohol		Cannabis		Nicotine		Latent substance use	
	*β* (95% CI)	*p* value	*β* (95% CI)	*p* value	*β* (95% CI)	*p* value	*β* (95% CI)	*p* value
Drinks per week-PGS	0.06 (0.01–0.10)	0.011	0.02 (−0.03 to 0.07)	0.397	0.06 (0.01–0.10)	0.018	0.06 (0.01–0.10)	0.014
Family history	0.27 (0.16–0.37)	5.5 × 10^−7^	0.33 (0.21–0.44)	2.4 × 10^−8^	0.46 (0.35–0.56)	5.9 × 10^−16^	0.52 (0.39–0.65)	9.8 × 10^−15^
Problematic alcohol use-PGS	0.10 (0.06–0.14)	7.8 × 10^−6^	0.06 (0.02–0.11)	0.010	0.09 (0.04–0.13)	3.6 × 10^−4^	0.10 (0.05–0.14)	2.5 × 10^−5^
Family history	0.24 (0.14–0.35)	4.6 × 10^−6^	0.31 (0.20–0.43)	1.3 × 10^−7^	0.44 (0.34–0.55)	2.4 × 10^−15^	0.50 (0.37–0.63)	8.4 × 10^−14^
Lifetime cannabis use-PGS	0.07 (0.02–0.11)	0.002	0.08 (0.03–0.13)	0.001	0.08 (0.04–0.13)	4.5 × 10^−4^	0.11 (0.06–0.15)	2.3 × 10^−6^
Family history	0.27 (0.17–0.37)	5.1 × 10^−7^	0.31 (0.20–0.43)	6.5 × 10^−9^	0.45 (0.34–0.56)	8.6 × 10^−16^	0.52 (0.39–0.65)	7.5 × 10^−15^
Cannabis use disorder-PGS	0.06 (0.02–0.11)	0.005	0.10 (0.05–0.14)	7.9 × 10^−5^	0.10 (0.05–0.15)	2.5 × 10^−5^	0.09 (0.05–0.14)	3.8 × 10^−5^
Family history	0.26 (0.16–0.36)	1.3 × 10^−6^	0.31 (0.20–0.42)	1.2 × 10^−7^	0.44 (0.33–0.55)	3.3 × 10^−15^	0.50 (0.37–0.63)	1.0 × 10^−13^
Regular smoking-PGS	0.09 (0.05–0.14)	1.9 × 10^−5^	0.12 (0.07–0.17)	8.6 × 10^−7^	0.17 (0.13–0.22)	1.2 × 10^−13^	0.15 (0.11–0.20)	2.1 × 10^−11^
Family history	0.26 (0.15–0.36)	1.5 × 10^−6^	0.3 (0.19–0.42)	2.2 × 10^−7^	0.42 (0.31–0.53)	3.2 × 10^−14^	0.48 (0.35–0.61)	3.6 × 10^−13^
Nicotine dependence-PGS	0.00 (−0.05 to 0.04)	0.845	0.00 (−0.05 to 0.05)	0.929	0.06 (0.02–0.11)	0.008	0.02 (−0.02 to 0.07)	0.294
Family history	0.27 (0.17–0.38)	4.4 × 10^−7^	0.33 (0.22–0.44)	2.1 × 10^−8^	0.46 (0.35–0.56)	3.7 × 10^−16^	0.53 (0.40–0.66)	6.6 × 10^−15^

*Notes.* Estimates in each panel are standardized betas with 95% CIs from separate linear mixed-effects models predicting scores on each substance use index between ages 14 and 24 as a function of each PGS and participant family history, adjusted for sex, zygosity, birth year, age at the most recent outcome assessment, and first 10 genetic PCs. ‘Family history’ in each model is matched to the outcome of interest (i.e. models shown in columns, from left to right, include a single binary variable representing the presence/absence of any family history of alcohol use disorder, family history of cannabis use disorder, family history of nicotine dependence, and family history of any SUD, respectively).

### To what extent are associations between PGSs capturing risk of substance use/SUDs and substance use in adolescence and young adulthood attributable to inherited genetic variation *v.* indirect effects?

Table 4 displays the within- and between-pair prediction estimates from models examining associations between each of the six PGSs and four substance use phenotypes using data from DZ twin pairs only. Significant associations were found for 21 of 24 (87.5%) between-pair estimates and only 4 of 24 (16.7%) within-pair estimates. On average, magnitudes of within-pair estimates (mean *β* *=* 0.04, range = −0.06 to 0.13) were about a quarter (74% reduction) of the magnitudes of significant between-pair estimates (mean *β* *=* 0.16, range = 0.05–0.26).

**Table 4.** Results from DZ-only co-twin analyses of each PGS and substance use in adolescence and adulthood

Polygenic score	Substance use outcome	Between-pair effect		Within-pair effect		Comparison	
		*β* (95% CI)	*p* value	*β* (95% CI)	*p* value	*Δ* (%)	*p* value
Drinks per week	Alcohol use	**0.17 (0.08–0.26)**	**4.1 × 10^−4^**	0.03 (−0.06 to 0.12)	0.480	82	0.028
	Cannabis use	**0.13 (0.03–0.23)**	**0.012**	0.01 (−0.10 to 0.12)	0.846	92	0.133
	Nicotine use	**0.12 (0.02–0.22)**	**0.019**	0.06 (−0.04 to 0.16)	0.258	50	2.7 × 10^−11^
	*Substance use factor*	**0.16 (0.07–0.26)**	**0.001**	0.04 (−0.05 to 0.13)	0.351	75	0.067
Problematic alcohol use	Alcohol use	**0.19 (0.10–0.28)**	**3.2 × 10^−5^**	0.01 (−0.08 to 0.10)	0.799	95	0.005
	Cannabis use	**0.15 (0.05–0.24)**	**0.003**	0.00 (−0.11 to 0.10)	0.948	100	0.017
	Nicotine use	**0.17 (0.08–0.26)**	**4.8 × 10^−4^**	0.03 (−0.07 to 0.13)	0.527	82	0.078
	*Substance use factor*	**0.20 (0.11–0.29)**	**2.5 × 10^−5^**	0.02 (−0.07 to 0.11)	0.684	90	0.005
Lifetime cannabis use	Alcohol use	0.07 (−0.02 to 0.17)	0.122	0.09 (0.00–0.18)	0.050	−28	0.790
	Cannabis use	**0.22 (0.13–0.32)**	**7.4 × 10^−6^**	0.08 (−0.03 to 0.19)	0.158	64	0.046
	Nicotine use	**0.12 (0.02–0.21)**	**0.018**	**0.13 (0.03–0.24)**	**0.011**	−8	0.823
	*Substance use factor*	**0.15 (0.06–0.25)**	**0.002**	**0.12 (0.03–0.22)**	**0.009**	20	0.657
Cannabis use disorder	Alcohol use	**0.12 (0.03–0.22)**	**0.011**	0.01 (−0.08 to 0.10)	0.847	92	0.083
	Cannabis use	**0.23 (0.13–0.33)**	**6.4 × 10^−6^**	0.03 (−0.07 to 0.14)	0.516	87	0.007
	Nicotine use	**0.19 (0.09–0.28)**	**2.6 × 10^−4^**	0.09 (−0.01 to 0.18)	0.079	53	0.137
	*Substance use factor*	**0.21 (0.11–0.30)**	**3.6 × 10^−5^**	0.06 (−0.03 to 0.14)	0.221	71	0.019
Regular smoking	Alcohol use	**0.14 (0.05–0.22)**	**0.003**	0.05 (−0.04 to 0.14)	0.252	64	0.159
	Cannabis use	**0.22 (0.13–0.32)**	**3.6 × 10^−6^**	0.07 (−0.03 to 0.18)	0.153	68	0.021
	Nicotine use	**0.26 (0.17–0.35)**	**3.3 × 10^−8^**	**0.14 (0.04–0.23)**	**0.006**	46	2.9 × 10^−20^
	*Substance use factor*	**0.25 (0.16–0.34)**	**1.6 × 10^−7^**	**0.11 (0.02–0.19)**	**0.017**	56	0.018
Nicotine dependence	Alcohol use	0.07 (−0.03 to 0.17)	0.184	−0.06 (−0.16 to 0.03)	0.176	186	0.048
	Cannabis use	0.05 (−0.05 to 0.16)	0.312	−0.03 (−0.14 to 0.08)	0.565	160	0.212
	Nicotine use	**0.15 (0.05–0.26)**	**0.004**	−0.05 (−0.15 to 0.06)	0.401	133	0.002
	*Substance use factor*	**0.12 (0.01–0.22)**	**0.027**	−0.06 (−0.15 to 0.04)	0.242	150	0.008

*Notes.* DZ-only co-twin control analyses decompose effects from individual-level PGS models into between-pair effects and within-pair effects. Estimates are reported as standardized betas, reflecting the s.d. increase on each measure of cumulative substance use with each s.d. increase in each PGS. The rightmost ‘Comparison’ column displays the percent reduction (*Δ*) of the within-pair effect relative to the between-pair effect, with associated *p* values indicating whether the difference in effects is statistically significant generated via likelihood ratio tests. The number of complete twin pairs used in all models is 339. Because standardization was conducted at the phenotypic level, betas for the within-pair effects should be interpreted in terms of the s.d. for the entire sample rather than the s.d. of twin differences. All models included participant age, sex, birth year, and the first 10 genetic PCs as covariates. CI = confidence interval; DZ = dizygotic. Between- and within-pair effects significantly different from zero (*p* < 0.05) are shown in bold.

Notably, significant differences in associations within and between twin pairs for PGSs predicting the use of the substance used to derive the indicator were found across all substance classes. Within-pair prediction was significantly lower than the between-pair prediction for the Drinks Per Week-PGS (*Δ* = 82%, *p* = 0.028), the Problematic Alcohol Use-PGS (*Δ* = 95%, *p* = 0.005), the Lifetime Cannabis Use-PGS (*Δ* = 64%, *p* = 0.046), the Cannabis Use Disorder-PGS (*Δ* = 87%, *p* = 0.007), the Regular Smoking-PGS (*Δ* = 46%, *p* = 2.9 × 10^−20^), and the Nicotine Use Disorder-PGS (*Δ* = 133%, *p* = 0.002). We observed similarly large (*Δ* ⩾ 50%) attenuations of within-pair relative to between-pair prediction for associations between PGSs and substances *not* used to derive the indicator, although these attenuations did not universally reach statistical significance (likely because the coefficients being compared tended to be smaller and less reliable). Taken together, this pattern of results suggests that the prediction effects of our six PGSs are likely mediated, in large part, by some combination of population stratification, assortative mating, and/or indirect genetic effects of parents. The only exceptions to this general pattern were found for PGSs capturing risk of Lifetime Cannabis Use and Regular Smoking, as within-pair associations between these scores and with both Nicotine and Latent Substance Use remained significant, suggesting direct effects of inherited genetic variation. Attenuation of within-pair *v.* between-pair estimates was particularly small (*Δ* ⩽ 20%) for associations between the Lifetime Cannabis Use-PGS and non-target substances (i.e. alcohol, nicotine, and latent substance use), suggesting these relationships were least inflated by indirect effects.

### Does behavioral disinhibition prior to substance use initiation account for the non-specific associations between risk indicators (i.e. PGSs and family history) and substance use in adolescence and young adulthood?

Consistent with prior research, twins with greater behavioral disinhibition at age 11 tended to also report greater alcohol [*β* (95% CI) = 0.13 (0.09–0.18), *p* *=* 2.0 × 10^−9^], cannabis [*β* (95% CI) = 0.16 (0.11–0.21), *p* *=* 2.8 × 10^−10^], and nicotine use [*β* (95% CI) = 0.26 (0.21–0.30), *p* *=* 2.1 × 10^−26^] in adolescence and adulthood. Twins with higher scores on each PGS tended to be more disinhibited than twins with lower PGS (although associations with the Lifetime Cannabis Use-PGS and Nicotine Dependence-PGS did not reach statistical significance). Family history of alcohol use disorder, cannabis use disorder, and nicotine dependence were also each associated with higher behavioral disinhibition (online Supplementary Table S4).

**Fig. 1.**
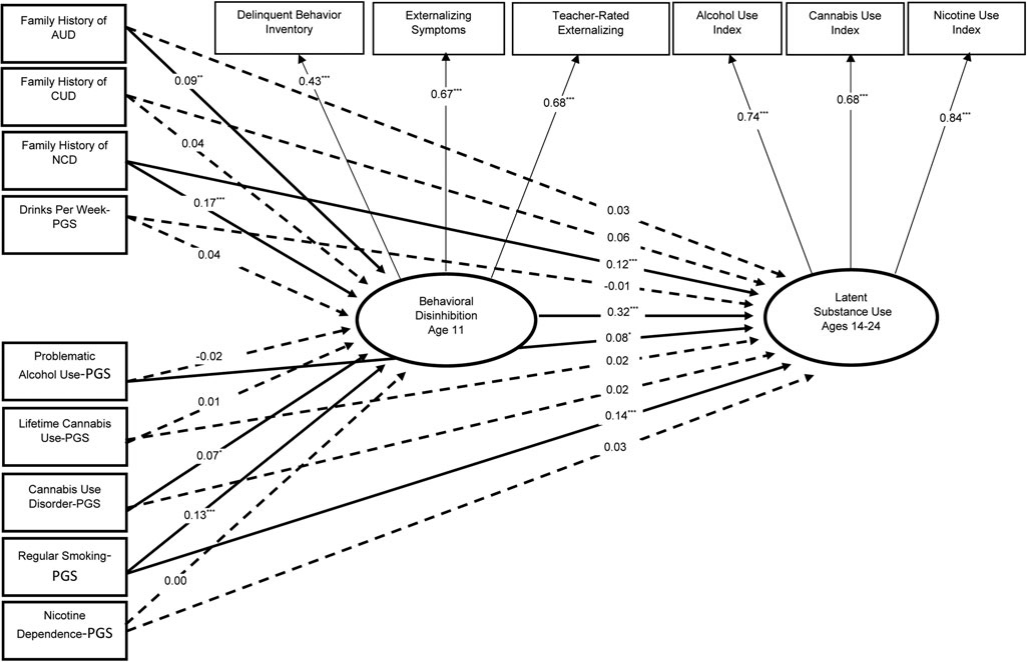
Structural equation model testing whether each risk indicator is independently associated with increased substance use in adolescence and young adulthood via increased behavioral disinhibition in preadolescence (*N* = 2483). *Notes.* Participants' age, sex, zygosity, birth year, and first 10 genetic PCs were included as covariates for both behavioral disinhibition and latent substance use. Paths for covariates are omitted in this figure for ease of display. Model fit was adequate: χ^2^ = 438.74, *p* < 0.001; CFI = 0.89; TLI = 0.84; RMSEA = 0.04; *R*^2^ latent substance use = 0.36. There were significant indirect paths from Family History of Alcohol Use Disorder [*β* (95% CI) = 0.03 (0.01–0.05), *p* = 0.012], Family History of Nicotine Dependence [*β* (95% CI) = 0.06 (0.03–0.09), *p* < 0.001], Regular Smoking-PGS [*β* (95% CI) = 0.04 (0.02–0.07), *p* = 0.001], and Cannabis Use Disorder-PGS [*β* (95% CI) = 0.02 (0.00–0.05), *p* = 0.033] to greater substance use via increased behavioral disinhibition. Corresponding indirect paths involving Family History of Cannabis Use Disorder [*β* (95% CI) = 0.01 (−0.01 to 0.04), *p* = 0.303], Drinks per Week-PGS [*β* (95% CI) = 0.01 (−0.01 to 0.04), *p* = 0.320], Lifetime Cannabis Use-PGS [*β* (95% CI) = 0.01 (−0.02 to 0.03), *p* = 0.695], Problematic Alcohol Use-PGS [*β* (95% CI) = −0.01 (−0.03 to 0.02), *p* = 0.613], and Nicotine Dependence-PGS [*β* (95% CI) = 0.00 (−0.02 to 0.02), *p* = 0.941] were nonsignificant. SUD = substance use disorder; PGS = polygenic score. Significant paths are shown as solid lines; nonsignificant paths (*p* > 0.05) are represented with dotted lines. *p < 0.05, **p < 0.01, ***p < 0.001.

Finally, we examined whether our PGS and family history measures were related to increased substance use via elevated behavioral disinhibition prior to substance use onset. Because each measure (other than the Nicotine Dependence-PGS) predicted the use of multiple types of substances, we estimated a latent substance use factor as our outcome in these mediation models. Results from individual-indicator models indicated significant indirect effects from all risk indicators (other than the Nicotine Dependence-PGS) to substance use between ages 14 and 24 via behavioral disinhibition at age 11 (online Supplementary Table S5). In the model including all nine indicators, only indirect paths for the Cannabis Use Disorder-PGS [*β* (95% CI) = 0.02 (0.00–0.05), *p* = 0.033], Regular Smoking-PGS [*β* (95% CI) = 0.04 (0.02–0.07), *p* = 0.001], family history of alcohol use disorder [*β* (95% CI) = 0.03 (0.01–0.05), *p* = 0.012], and family history of nicotine dependence [*β* (95% CI) = 0.06 (0.03–0.09), *p* < 0.001] remained significant, indicating that each measure was independently associated with substance use via elevated behavioral disinhibition. Conversely, the indirect paths from the Drinks Per Week-PGS, Problematic Alcohol Use-PGS, Lifetime Cannabis Use-PGS, and family history of cannabis use disorder to latent substance use via behavioral disinhibition were reduced to near-zero, likely due to the weaker associations between these measures and our latent disinhibition factor. Significant direct effects from the Problematic Alcohol-PGS, the Regular Smoking-PGS, and family history of nicotine dependence to substance use suggest that these measures additionally contribute to substance use via mechanisms that do not involve behavioral disinhibition (Fig. 1).

## Discussion

Results from this longitudinal study of substance use advance the understanding of recently developed PGSs capturing risk of substance use/SUDs in three ways. First, we showed that all six PGSs remained associated with at least one substance use index after adjusting for family history of the corresponding SUD, suggesting independent effects. Second, we contrasted within- and between-pair PGS effects to show that PGS prediction is substantially driven by the effects of demography and indirect genetic effects of parents. Third, longitudinal mediation analyses indicated that multiple PGS and family history measures contributed to substance use in adolescence and adulthood via elevated behavioral disinhibition in preadolescence, highlighting a possible developmental pathway that might partly explain the nonspecific associations commonly observed between these indicators and multiple types of substance use.

Strengths of the current study include the multiple approaches to estimating risk and different types of substance use assessed, allowing us to examine the consistency of associations between these measures as well as test for independent effects. Our finding that all PGS and family history measures were associated with the use of multiple substances is in line with previous studies (e.g. Chang et al., 2019; Deak et al., 2020; Merikangas et al., 1998). However, we extend previous research by showing that all PGS associations remained significant even after adjusting for the corresponding family history measures. We expect family history will continue to outperform PGSs in the prediction of future substance use given that PGSs assume an additive model of effects and capture only common genetic variants, limiting their predictive power (Choi et al., 2020). Nevertheless, because our results suggest that family history and PGS measures may tap different elements of risk, it is possible that PGSs for substance use/SUDs may still offer incremental predictive utility in research and clinical settings, particularly as substance use phenotypes are refined further and GWAS discovery samples continue to grow.

Our finding that within-pair associations between PGSs and substance use outcomes were generally small, nonsignificant, and significantly smaller than the corresponding between-pair associations highlights the influence of demographic and family environmental factors on substance use in adolescence and young adulthood. Between-family PGS prediction of substance use may thus be substantially driven by these effects. These results are compatible with research suggesting that PGS prediction of other complex psychological phenotypes (e.g. IQ and educational attainment) is also substantially driven by these indirect processes (Selzam et al., 2019). However, significant within-pair associations between two PGSs (i.e. the Lifetime Cannabis Use-PGS and Regular Smoking-PGS) and nicotine use indicate that the corresponding between-pair associations cannot be fully explained by these processes, suggesting a direct genetic effect that may be unique to these measures.

Our finding that higher scores on multiple PGS and family history measures were associated with greater behavioral disinhibition at age 11 extends previous findings from this sample indicating that the Regular Smoking-PGS indexes broad genetic risk for multiple types of substance use and externalizing disorders (Deak et al., 2020; Hicks et al., 2021). The weaker associations with the Lifetime Cannabis Use-PGS and Nicotine Dependence-PGS that we observed could be attributable to a smaller number of pleiotropic alleles in these PGSs relative to the other polygenic risk measures, or simply to differences in sample size and heritability in the discovery GWASs used to develop each score (see Table 2). Results from our mediation models are consistent with previous findings documenting substantial latent genetic overlap between contemporaneously assessed behavioral disinhibition and substance use in late adolescence (e.g. Vrieze, McGue, Miller, Hicks, & Iacono, 2013). We build upon these findings here by showing that (a) this association extends to contemporary risk indicators (e.g. PGSs and family history measures) and (b) ruling out the possibility of reverse-causation (i.e. that substance use contributes to elevations in behavioral disinhibition) by testing for associations with behavioral disinhibition measured prior to substance use onset.

Nevertheless, we acknowledge limitations. First, because our analyses were restricted to participants of European ancestry, it is unclear if observed PGS associations will generalize to other ancestral groups with different allele frequencies (Duncan et al., 2019). Second, it is possible that some important within-pair effects did not reach statistical significance in our within-twin pair models due to insufficient statistical power. It is, therefore, possible that analyses conducted in larger DZ twin samples or using more powerful PGSs may find greater evidence of direct genetic effects. Third, our analyses were right-hand censored at age 24. We focused on substance use during adolescence and early adulthood because (a) these are the developmental periods of peak substance use, and (b) research suggests that behavioral disinhibition is a predictor of early-onset substance use, in particular (Iacono et al., 2008). However, this focus on early life means that the relationships between our measures of risk, substance use, and behavioral disinhibition in later life are unclear, suggesting it will be important to continue following genotyped cohorts with prospective assessments of substance use into middle and old age. Finally, although the temporal ordering of our behavioral disinhibition and substance use assessments allows us to rule out the possibility of reverse-causation, observational research cannot establish whether the mediation we observed is causal. Future research will, therefore, be needed to assess whether interventions that modify behavioral disinhibition in individuals at different levels of risk reduce subsequent substance use.

Despite these limitations, several implications can be drawn from this study. Although we show that PGSs capturing risk of substance use/SUDs can be combined with family history data to augment prediction of substance use in adolescence and young adulthood, results from within-twin pair models indicate that much of this augmentation likely comes from indirect processes. This observation suggests that interventions targeting individuals at high polygenic risk of substance use/SUDs may benefit from a focus on family level factors. In addition, our results buttress previous reports suggesting that many existing substance- or disorder-specific measures of polygenic risk also tap transdiagnostic risk factors (e.g. disinhibition) (Hicks et al., 2021; Waszczuk et al., 2021), lending support to ongoing efforts aimed at quantifying the risk of these higher-order phenotypes using molecular genetic data (e.g. Linnér et al., 2021) and raising the possibility that PGSs derived from these efforts may further enhance prediction of substance use. Finally, our results support using elevated behavioral disinhibition in preadolescence to aid in the identification of at-risk youth, similar to existing interventions that target adolescents with elevated scores on measures of sensation-seeking or impulsivity (e.g. Conrod, Stewart, Comeau, & Maclean, 2006, 2010). It is our hope that research focused on the polygenic prediction of substance use will facilitate the identification of vulnerable youth before substance exposure, potentially setting the stage for targeted interventions that avert harmful patterns of behavior several years before they onset.

## Footnotes

† The notes appear after the main text.

## Footnotes

1 The specific DSM-III-R and DSM-5 substance use disorder criteria are comparable to one another with the exception that DSM-III-R includes a symptom of recurrent legal problems that is not included in DSM-5, and DSM-5 includes a symptom of craving that is not included in DSM-III-R (Robinson & Adinoff, 2016).

2 We included only DZ twins in these models because genetically identical MZ twins have zero within-pair variation in their polygenic scores.

## Supplementary material

For supplementary material accompanying this paper visit https://doi.org/10.1017/S0033291721004116. click here to view supplementary material
