# Supplementary material for: Associations between polygenic risk of substance use and use disorder and alcohol, cannabis, and nicotine use in adolescence and young adulthood in a longitudinal twin study
Source: Psychol Med. 2021 Oct 12;53(6):2296–306. doi: 10.1017/S0033291721004116 (PMC10123833; doi:10.1017/S0033291721004116)
Supplement: Supplementary file 1 [file S0033291721004116sup001.docx]

**Supplementary Material for the Article:**

**Associations between polygenic risk of substance use and use disorder and alcohol, cannabis, and nicotine use in adolescence and young adulthood
in a longitudinal twin study**

Jonathan D. Schaefer, Ph.D,^1^ Seon-Kyeong Jang, M.A.,^2^ D. Angus Clark, Ph.D,^3^

Joseph D. Deak, Ph.D,^4,5^ Brian M. Hicks, Ph.D,^3^ William G. Iacono, Ph.D,^2^

Mengzhen Liu, Ph.D,^2^ Matt McGue, Ph.D,^2^ Scott I. Vrieze, Ph.D,^2^ Sylia Wilson, Ph.D^1^

^1^ Institute for Child Development, University of Minnesota, Minneapolis, MN, USA

^2^ Department of Psychology, University of Minnesota, Minneapolis, MN, USA

^3^ Department of Psychiatry, University of Michigan
^4^Department of Psychiatry, Yale University School of Medicine
^5^Department of Psychiatry, Veterans Affairs Connecticut Healthcare Center

**This material supplements, but does not replace, the peer-reviewed paper in**

***Psychological Medicine***

**Correspondence:**

Jonathan D. Schaefer, Ph.D
Institute of Child Development, University of Minnesota
51 E. River Rd., Minneapolis, MN 55455
schae567@umn.edu

**Table S1.** Items used in substance use indices.

|  | **Alcohol** | | | |  | | **Cannabis** | |  | **Nicotine** |
| --- | --- | --- | --- | --- | --- | --- | --- | --- | --- | --- |
| **Score** | **Frequency** | **Amount**  **(# of drinks)** | **Max Drinks** | **Intoxications** | |  | **Frequency** | **Amount**  **(# of uses)** |  | **Cigarettes per day** |
| 0 | None | 0 | 0 | 0 | |  | None | 0 |  | None |
| 1 | Less than once per year to less than once a month | 1 ­– 3 | 1 – 3 | 1 – 5 | |  | Less than once per year to less than once a month | 1 – 4 |  | 1 – 2 |
| 2 | 1-3 times per month | 4 – 6 | 4 – 6 | 6 – 10 | |  | 1-3 times per month | 5 – 30 |  | 3 – 9 |
| 3 | 1-4 times per week | 7 – 10 | 7 – 10 | 11 – 20 | |  | 1-4 times per week | 31 – 100 |  | 10^*^ – 19 |
| 4 | Nearly every day to once a day | 11 – 20 | 11 – 20 | 21 – 50 | |  | Nearly every day to once a day | 101 – 400 |  | ≥ 20^†^ |
| 5 | Two or more times a day | 21 – 29 | 21 – 29 | 51 – 149 | |  | Two or more times a day | ≥ 401 |  | - |
| 6 | - | ≥ 30 | ≥ 30 | ≥ 150 | |  | - | - |  | - |

*Notes.* Cannabis amount and alcohol/cannabis frequency items were ordinalized to a 0-5 scale, cigarettes per day to a 0-4 scale, and the remaining items to a 0-6 scale.
*or half a pack
†or a pack or more

**Figure S1.** Zero-order correlations among risk indicator variables, behavioral disinhibition mediator variables, and substance use outcome variables.

**
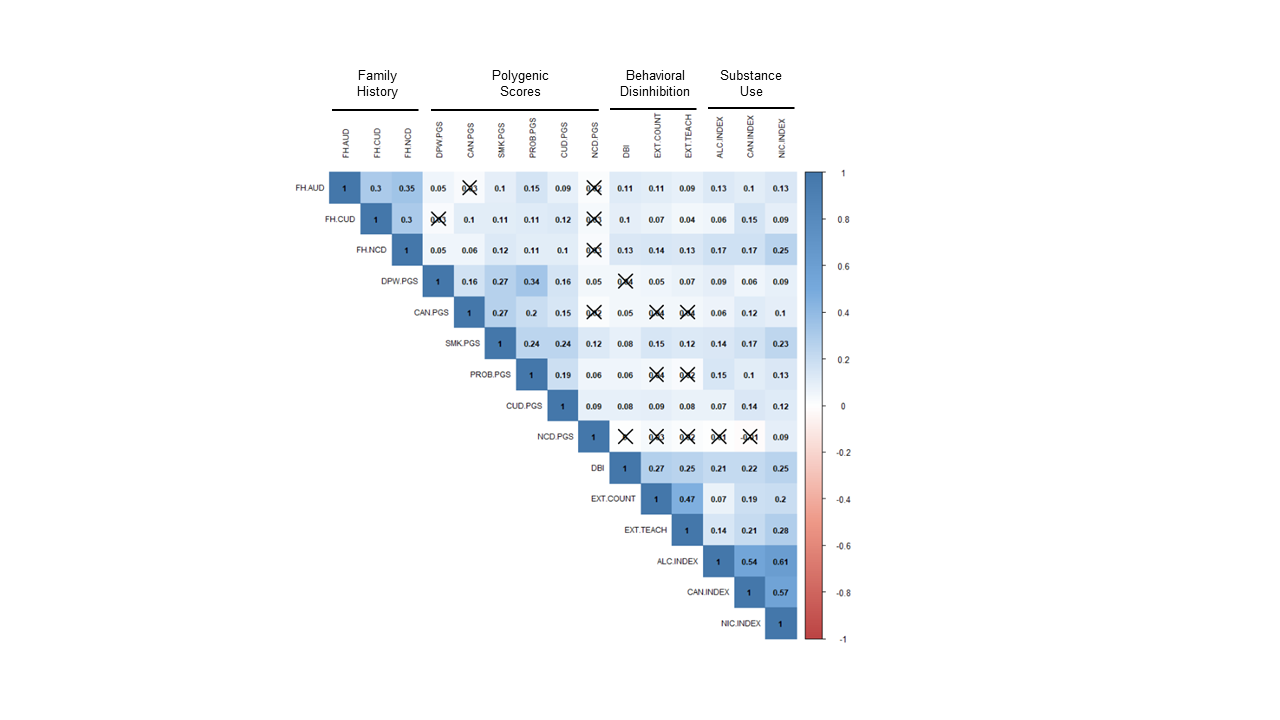
**

*Notes.* Figure displays zero-order correlations among study variables, with non-significant correlations indicated with a superimposed “X”. FH.AUD = family history of alcohol use disorder, FH.CUD = family history of cannabis use disorder, FH.NCD = family history of nicotine dependence, DPW.PGS = Drinks Per Week-PGS, CAN.PGS = Lifetime Cannabis Use-PGS, SMK.PGS = Regular Smoking-PGS, PROB.PGS = Problematic Alcohol Use-PGS, CUD.PGS = Cannabis Use Disorder-PGS, NCD.PGS = Nicotine Dependence-PGS, DBI = Delinquent Behavior Inventory scores, EXT.COUNT = count of Externalizing symptoms, EXT.TEACH = teacher reported-externalizing problems, ALC.Index = alcohol use index, CAN.Index = cannabis use index, NIC.Index = nicotine use index.

**Table S3.** Associations between each risk indicator and substance use index.

|  | **Substance Use Indices (Ages 14-24)** | | | | | | | | | | | | | | | | |
| --- | --- | --- | --- | --- | --- | --- | --- | --- | --- | --- | --- | --- | --- | --- | --- | --- | --- |
|  | **Alcohol** | |  | | **Cannabis** | | |  | | **Nicotine** | | |  | | **Substance Use Factor** | | |
| **Predictors** | **β  (95% CI)** | ***p*-value** | |  | | **β**  **(95% CI)** | ***p*-value** | |  | | **β (95% CI)** | ***p*-value** | |  | | **β (95% CI)** | ***p*-value** |
| **Polygenic Scores (PGSs)** |  |  | |  | |  |  | |  | |  |  | |  | |  |  |
| *Drinks Per Week-PGS* | 0.06  (0.02, 0.11) | .006 | |  | | 0.04  (-0.01, 0.08) | .155 | |  | | 0.07  (0.02, 0.11) | .006 | |  | | 0.06  (0.02, 0.11) | .005 |
| *Problematic Alcohol Use-PGS* | 0.11  (0.07, 0.15) | 1.0 x 10^-6^ | |  | | 0.08  (0.03, 0.13) | 8.5 x 10^-4^ | |  | | 0.10  (0.06, 0.15) | 2.2 x 10^-5^ | |  | | 0.11  (0.07, 0.16) | 1.2 x 10^-6^ |
| *Lifetime Cannabis Use-PGS* | 0.08  (0.03, 0.12) | .001 | |  | | 0.11  (0.07, 0.16) | 2.7 x 10^-6^ | |  | | 0.11  (0.06, 0.15) | 1.1 x 10^-5^ | |  | | 0.12  (0.07, 0.16) | 4.2 x 10^-7^ |
| *Cannabis Use Disorder-PGS* | 0.07  (0.02, 0.11) | .002 | |  | | 0.11  (0.07, 0.16) | 2.9 x 10^-6^ | |  | | 0.12  (0.07, 0.16) | 8.1 x 10^-7^ | |  | | 0.11 (0.07, 0.16) | 7.3 x 10^-7^ |
| *Regular Smoking-PGS* | 0.10  (0.06, 0.14) | 6.1 x 10^-6^ | |  | | 0.15  (0.10, 0.19) | 1.8 x 10^-9^ | |  | | 0.20  (0.15, 0.24) | 3.0 x 10^-17^ | |  | | 0.17  (0.13, 0.22) | 1.1 x 10^-14^ |
| *Nicotine Dependence-PGS* | 0.00 (-0.04, 0.05) | .959 | |  | | 0.00 (-0.05, 0.05) | .998 | |  | | 0.07 (0.02, 0.12) | .004 | |  | | 0.02  (-0.02, 0.08) | .203 |
| **Family History** |  |  | |  | |  |  | |  | |  |  | |  | |  |  |
| *Alcohol Use Disorder* | 0.29 (0.19, 0.39) | 1.8 x 10^-8^ | |  | | 0.21 (0.10, 0.32) | 1.7 x 10^-4^ | |  | | 0.25 (0.15, 0.35) | 2.9 x 10^-6^ | |  | | 0.29 (0.19, 0.40) | 4.3 x 10^-8^ |
| *Cannabis Use Disorder* | 0.22 (0.11, 0.32) | 5.9 x 10^-5^ | |  | | 0.38 (0.27, 0.49) | 2.2 x 10^-11^ | |  | | 0.31 (0.20, 0.42) | 1.5 x 10^-8^ | |  | | 0.35 (0.24, 0.45) | 3.0 x 10^-10^ |
| *Nicotine Dependence* | 0.33 (0.23, 0.43) | 1.5 x 10^-10^ | |  | | 0.32 (0.21, 0.43) | 1.0 x 10^-10^ | |  | | 0.47 (0.37, 0.57) | 2.1 x 10^-18^ | |  | | 0.45 (0.34, 0.55) | 8.5 x 10^-17^ |

*Notes.* Each standardized beta (with 95% confidence interval) and corresponding p-value are from separate linear mixed-effects models predicting scores on each substance use index between ages 14-24 as a function of each individual risk indicator, adjusting for participant sex, zygosity, birth year, and age at the most recent outcome assessment. Models including polygenic scores (PGSs) were further adjusted for the first 10 genetic principal components. Model Ns ranged from 1597 to 2031.

**Table S4.** Associations between each risk indicator and behavioral disinhibition.

|  | | **Behavioral Disinhibition (Age 11)** | |
| --- | --- | --- | --- |
| **Predictors** | **β  (95% CI)** | | ***p*-value** |
| **Polygenic Scores (PGSs)** |  | |  |
| *Drinks Per Week-PGS* | 0.08 (0.03, 0.12) | | .003 |
| *Problematic Alcohol Use-PGS* | 0.06 (0.01, 0.11) | | .022 |
| *Lifetime Cannabis Use-PGS* | 0.04 (-0.01, 0.09) | | .114 |
| *Cannabis Use Disorder-PGS* | 0.08 (0.03, 0.13) | | .001 |
| *Regular Smoking-PGS* | 0.12 (0.07, 0.17) | | 1.4 x 10^-6^ |
| *Nicotine Dependence-PGS* | 0.02 (-0.03, 0.07) | | .370 |
| **Family History** |  | |  |
| *Alcohol Use Disorder* | 0.27 (0.16, 0.38) | | 1.1 x 10^-6^ |
| *Cannabis Use Disorder* | 0.23 (0.11, 0.34) | | 7.1 x 10^-5^ |
| *Nicotine Dependence* | 0.35 (0.25, 0.46) | | 8.6 x 10^-11^ |

*Notes.* Each standardized beta (with 95% confidence interval) and corresponding p-value are from separate linear mixed-effects models predicting scores on the behavioral disinhibition factor at age 11 as a function of each individual risk indicator, adjusting for participant sex, zygosity, birth year, and age at the most recent outcome assessment. Models including polygenic scores (PGSs) were further adjusted for the first 10 genetic principal components. Model Ns ranged from 1751 to 1998.

**Table S5.** Path estimates from separate mediation models testing whether each risk indicator is associated with increased substance use in adolescence and young adulthood via increased behavioral disinhibition in preadolescence (all Ns = 2483).

| **Model** | **Standardized path coefficients  (95% CI)** | | **p-value** | |
| --- | --- | --- | --- | --- |
| **Family History** |  |  | |  |
| *Family History of Alcohol Use Disorder (FH-AUD) Model* |  |  | |  |
| FH-AUD 🡪 Behavioral Disinhibition age 11 | 0.18 (0.12, 0.23) | <.001 | |  |
| Behavioral Disinhibition age 11 🡪 Substance Use ages 14-24 | 0.39 (0.31, 0.47) | <.001 | |  |
| Direct Effect: FH-AUD 🡪 Substances Use ages 14-24 | 0.10 (0.04, 0.15) | .001 | |  |
| Indirect Effect: FH-AUD 🡪 Behavioral Disinhibition 🡪 Substance Use | 0.07 (0.04, 0.10) | <.001 | |  |
| Total Effect | 0.16 (0.11, 0.22) | <.001 | |  |
| *Family History of Cannabis Use Disorder (FH-CUD) Model* |  |  | |  |
| FH-CUD 🡪 Behavioral Disinhibition age 11 | 0.14 (0.07, 0.20) | <.001 | |  |
| Behavioral Disinhibition age 11 🡪 Substance Use ages 14-24 | 0.39 (0.31, 0.47) | <.001 | |  |
| Direct Effect: FH-CUD 🡪 Substances Use ages 14-24 | 0.13 (0.07, 0.19) | <.001 | |  |
| Indirect Effect: FH-CUD 🡪 Behavioral Disinhibition 🡪 Substance Use | 0.05 (0.03, 0.08) | <.001 | |  |
| Total Effect | 0.13 (0.12, 0.24) | <.001 | |  |
| *Family History of Nicotine Dependence (FH-NCD) Model* |  |  | |  |
| FH-NCD 🡪 Behavioral Disinhibition age 11 | 0.24 (0.19, 0.30) | <.001 | |  |
| Behavioral Disinhibition age 11 🡪 Substance Use ages 14-24 | 0.36 (0.28, 0.44) | <.001 | |  |
| Direct Effect: FH-NCD 🡪 Substances Use ages 14-24 | 0.17 (0.11, 0.22) | <.001 | |  |
| Indirect Effect: FH-NCD 🡪 Behavioral Disinhibition 🡪 Substance Use | 0.09 (0.06, 0.12) | <.001 | |  |
| Total Effect | 0.26 (0.21, 0.30) | <.001 | |  |
| **Polygenic Scores** |  |  | |  |
| *Drinks Per Week-PGS Model* |  |  | |  |
| Drinks Per Week-PGS 🡪 Behavioral Disinhibition age 11 | 0.09 (0.03, 0.16) | .005 | |  |
| Behavioral Disinhibition age 11🡪 Substance Use ages 14-24 | 0.40 (0.33, 0.48) | <.001 | |  |
| Direct Effect: Drinks Per Week-PGS 🡪 Substance Use ages 14-24 | 0.07 (0.01, 0.12) | .021 | |  |
| Indirect Effect: Drinks Per Week-PGS 🡪 Behavioral Disinhibition 🡪 Substance Use | 0.04 (0.01, 0.07) | .008 | |  |
| Total Effect | 0.10 (0.05, 0.16) | <.001 | |  |
| *Problematic Alcohol Use-PGS Model* |  |  | |  |
| Problematic Alcohol Use-PGS 🡪 Behavioral Disinhibition age 11 | 0.08 (0.01, 0.14) | .021 | |  |
| Behavioral Disinhibition age 11🡪 Substance Use ages 14-24 | 0.40 (0.32, 0.47) | <.001 | |  |
| Direct Effect: Problematic Alcohol Use-PGS 🡪 Substance Use ages 14-24 | 0.15 (0.09, 0.21) | <.001 | |  |
| Indirect Effect: Problematic Alcohol Use-PGS 🡪 Behavioral Disinhibition 🡪 Substance Use | 0.03 (0.01, 0.06) | .029 | |  |
| Total Effect | 0.18 (0.12, 0.24) | <.001 | |  |
| *Lifetime Cannabis Use-PGS Model* |  |  | |  |
| Lifetime Cannabis Use-PGS 🡪Behavioral Disinhibition age 11 | 0.08 (0.03, 0.15) | .029 | |  |
| Behavioral Disinhibition age 11🡪 Substance Use ages 14-24 | 0.40 (0.33, 0.48) | <.001 | |  |
| Direct Effect: Lifetime Cannabis Use-PGS 🡪 Substance Use ages 14-24 | 0.09 (0.03, 0.15) | .003 | |  |
| Indirect Effect: Lifetime Cannabis Use-PGS 🡪 Behavioral Disinhibition 🡪 Substance Use | 0.03 (0.00, 0.06) | .035 | |  |
| Total Effect | 0.12 (0.06, 0.18) | <.001 | |  |
| *Cannabis Use Disorder-PGS Model* |  |  | |  |
| Cannabis Use Disorder-PGS 🡪 Behavioral Disinhibition age 11 | 0.14 (0.08, 0.20) | <.001 | |  |
| Behavioral Disinhibition age 11🡪 Substance Use ages 14-24 | 0.40 (0.32, 0.47) | <.001 | |  |
| Direct Effect: Cannabis Use Disorder-PGS 🡪 Substance Use ages 14-24 | 0.09 (0.04, 0.15) | .001 | |  |
| Indirect Effect: Cannabis Use Disorder-PGS 🡪 Behavioral Disinhibition 🡪 Substance Use | 0.06 (0.03, 0.08) | <.001 | |  |
| Total Effect | 0.15 (0.09, 0.20) | <.001 | |  |
| *Regular Smoking-PGS Model* |  |  | |  |
| Regular Smoking-PGS 🡪 Behavioral Disinhibition age 11 | 0.19 (0.13, 0.25) | <.001 | |  |
| Behavioral Disinhibition age 11🡪 Substance Use ages 14-24 | 0.37 (0.29, 0.45) | <.001 | |  |
| Direct Effect: Regular Smoking-PGS 🡪 Substance Use ages 14-24 | 0.19 (0.13, 0.24) | <.001 | |  |
| Indirect Effect: Regular Smoking-PGS 🡪 Behavioral Disinhibition 🡪 Substance Use | 0.07 (0.04, 0.10) | <.001 | |  |
| Total Effect | 0.26 (0.20, 0.31) | <.001 | |  |
| *Nicotine Dependence-PGS Model* |  |  | |  |
| Nicotine Dependence-PGS 🡪 Behavioral Disinhibition age 11 | 0.03 (-0.04, 0.09) | .395 | |  |
| Behavioral Disinhibition age 11🡪 Substance Use ages 14-24 | 0.41 ( 0.33, 0.49) | <.001 | |  |
| Direct Effect: Nicotine Dependence-PGS 🡪 Substance Use ages 14-24 | 0.06 ( 0.00, 0.12) | .063 | |  |
| Indirect Effect: Nicotine Dependence-PGS 🡪 Behavioral Disinhibition 🡪 Substance Use | 0.01 (-0.01, 0.04) | .404 | |  |
| Total Effect | 0.07 ( 0.01, 0.13) | .028 | |  |

*Note.* Each mediation model includes a single risk indicator. Participants’ age, sex, zygosity, and birth year were included as covariates for both behavioral disinhibition and latent substance use. Models using polygenic scores included the first 10 genetic principal components as additional covariates. The fit of each model was adequate (Family History of Alcohol Use Disorder: χ^2^ = 279.22, p<.001, CFI = 0.90, TLI = 0.85, RMSEA = 0.05; Family History of Cannabis Use Disorder: χ^2^ = 288.81, p<.001, CFI = 0.90, TLI = 0.84, RMSEA = 0.06; Family History of Nicotine Use Disorder: χ^2^ = 285.82, p<.001, CFI = 0.90, TLI = 0.85, RMSEA = 0.06; Drinks Per Week-PGS: χ^2^ = 353.01, p<.001, CFI = 0.90, TLI = 0.84, RMSEA = 0.04; Problematic Alcohol Use-PGS: χ^2^ = 359.22, p<.001, CFI = 0.90, TLI = 0.84, RMSEA = 0.04; Lifetime Cannabis Use-PGS: χ^2^ = 355.99, p<.001, CFI = 0.90, TLI = 0.84, RMSEA = 0.04; Cannabis Use Disorder-PGS: χ^2^ = 358.69, p<.001, CFI = 0.90, TLI = 0.84, RMSEA = 0.04; Regular Smoking-PGS: χ^2^ = 358.09, p<.001, CFI = 0.90, TLI = 0.85, RMSEA = 0.04; Nicotine Dependence-PGS: χ^2^ = 370.41, p<.001, CFI = 0.89, TLI = 0.84, RMSEA = 0.04). 95% confidence intervals derived via bias-corrected bootstrap procedure with 10,000 random draws. PGS = polygenic score. ^*^ p < .05, ^**^ p < .01, ^***^ p < .001.
